# On the Hunt for the Histone Code

**DOI:** 10.1016/j.mcpro.2024.100873

**Published:** 2024-11-01

**Authors:** Beatrix M. Ueberheide, Sahana Mollah, Benjamin A. Garcia

**Affiliations:** 1 Proteomics Laboratory, Division of Advanced Research Technologies, Department of Biochemistry and Molecular Pharmacology, New York University Langone Health Center, New York, New York, USA; 2 Department of Neurology, New York University Langone Health Center, New York, New York, USA; 3 SCIEX, Redwood City, California, USA; 4 Department of Biochemistry and Molecular Biophysics, Washington University School of Medicine, St Louis, Missouri, USA

**Keywords:** histone, epigenetics, proteomics, mass spectrometry, post-translational modifications

## Abstract

Our genome is not made of naked DNA but a fiber (chromatin) composed of DNA and proteins packaged into our chromosomes. The basic building block of chromatin is the nucleosome, which has two copies of each of the proteins called histones (H2A, H2B, H3, and H4) wrapped by 146 base pairs of DNA. Regions of our genetic material are found between the more open (euchromatin) and more compact (heterochromatin) regions of the genome that can be variably accessible to the underlying genes. Furthermore, post-translational modifications (PTMs) on histones, such as on H3, are critical for regulating chromatin accessibility and gene expression. While site-specific antibodies were the tool of choice for histone PTM analysis in the early days (pre-2000s), enter Don Hunt changing the histone PTM field forever. Don’s clever thinking brought new innovative mass spectrometry-based approaches to the epigenetics field. His lab’s effort led to the discovery of many new histone modifications and methods to facilitate the detection and quantification of histone PTMs, which are still considered state of the art in the proteomics field today. Due to Don’s pioneering work in this area, many labs have been able to jump into the epigenetics field and “Hunt” down their own histone targets. A walkthrough of those early histone years in the Hunt Lab is described by three of us who were fortunate enough to be at the right place, at the right time.

## Highlights

• Early mass spectrometry approaches were developed for analyzing histone modifications.
• Propionylation chemical derivatization proposed by Don Hunt used to facilitate the analysis of histone proteins by mass spectrometry.
• Many novel histone modifications and combinations of modifications were identified by the Hunt lab in the early 2000s.
• In collaboration with many labs, the Hunt lab helped quantify histone modifications across many biological processes.

## Graphical Abstract

**Figure:**
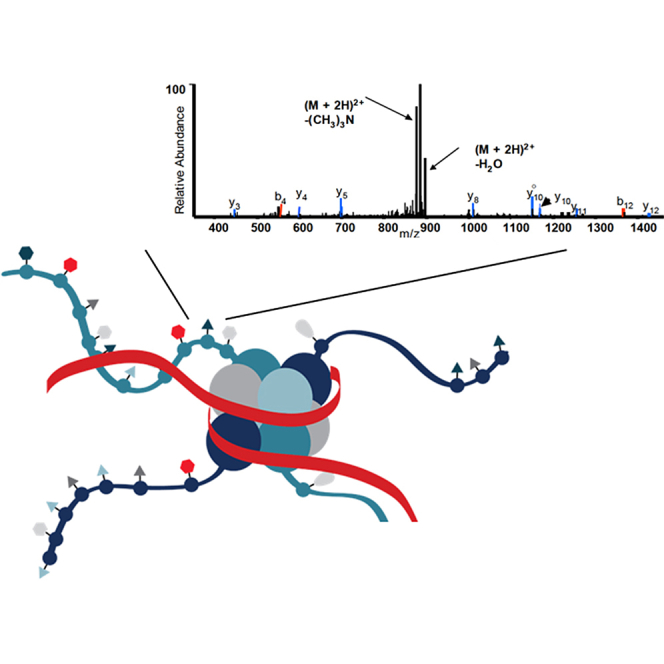

## In Brief

In the early 2000s before high resolution mass spectrometers were readily available, the Hunt lab developed many approaches to analyze histone modifications on low resolution instruments.

Histone proteins are part of chromatin, the dynamic structure of DNA and proteins that make up the chromosomes. The basic repeating unit of chromatin, the nucleosome, is a segment of DNA wrapped around two copies each of histone H2A, H2B, H3, and H4, the so-called core histones (1). These histone proteins can be modified via post-translational modifications (PTMs) that, in turn, change the structure of chromatin and provide anchor points for recruiting various proteins with different functionalities that further modify the chromatin. Thus, histone proteins and their PTMs make them key regulators of chromatin function and allow the chromatin polymer to be flexible so that the underlying genetic information can be accessed in a precisely regulated manner (Histone Code) (2, 3). Mass spectrometry has played a fundamental role in characterizing the various histone PTMs and their role in chromatin regulation (4, 5, 6, 7, 8, 9). Early on, mass spectrometry was demonstrated to be a powerful tool to monitor the molecular weight distributions of intact histone proteins, identifying PTMs that could be altered upon drug treatment (e.g. histone deacetylase inhibitor, HDACi) (7). While there have been many mass spectrometry labs that have contributed to histone modification analyses, Don Hunt and his lab have hunted down histone PTMs for a sustained long time. Back in the early '90s, this started when Don teamed up with Juan Ausio, and they kicked off a series of pioneering studies (10, 11). These early collaborations not only dug deep into the role of H1-like proteins in organizing sperm chromatin, but also marked one of the first times mass spectrometry was used to explore histone post-translational modifications. It was a big step forward in the field for both histones and mass spectrometry, a collaboration that has continued for decades (10, 11).

## A Partnership Made in Histone Heaven

**Fig. 1:**
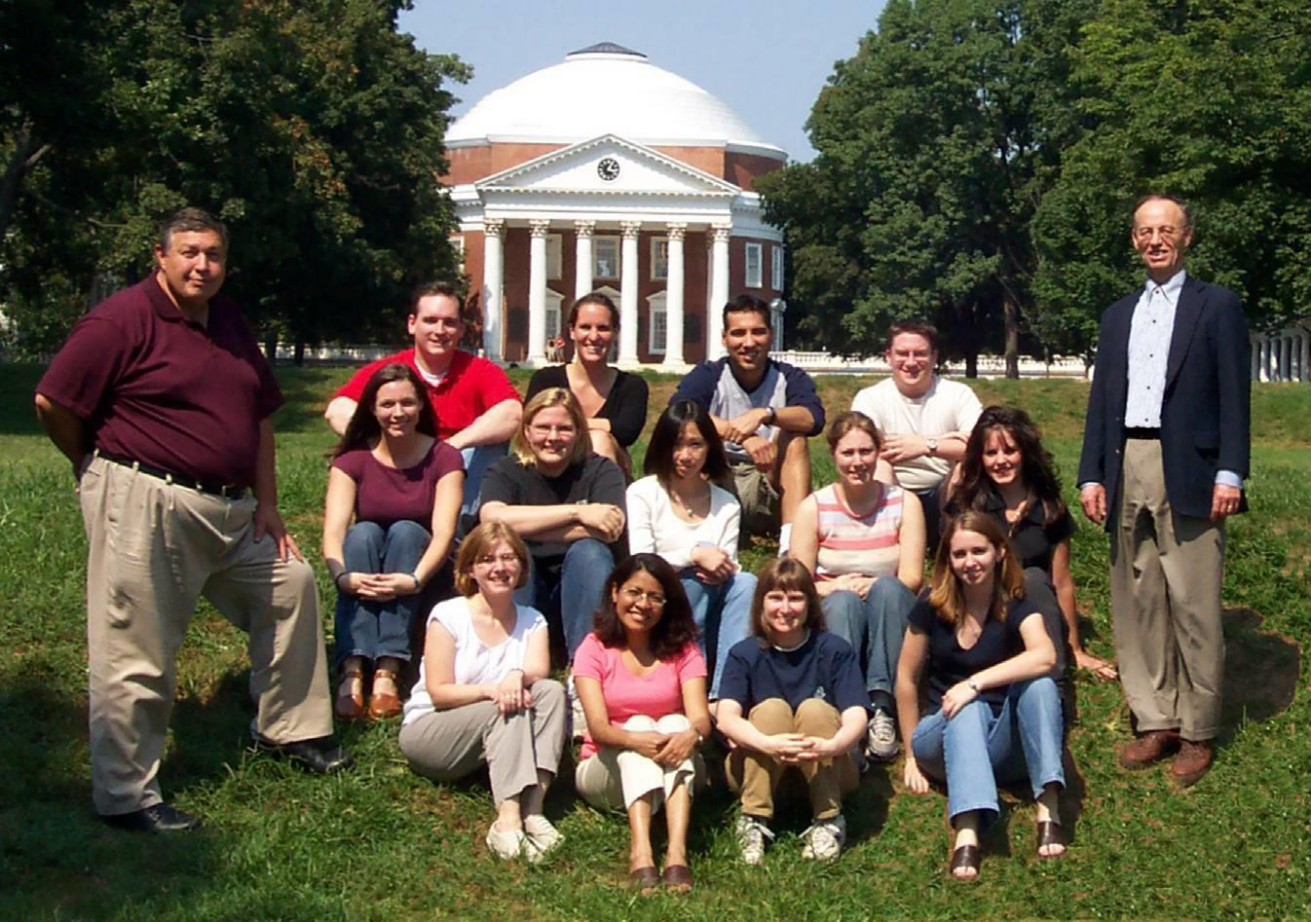
**Professor Donald F. Hunt and Dr. Jeffrey Shabanowitz and all the Hunt-lings (circa ∼2004).***Front row*: Erin Jeffery, **Sahana Mollah**, Dina Bai and Melanie Schroeder. *Second row*: Jeff Shabanowitz, Joy Polefrone, Annie Evans, An Chi, **Celeste Ptak**, Leann Mikesh and Don Hunt. *Third row*: Mark Platt, **Trixi Ueberheide**, **Ben Garcia** and **Scott Busby**. (In *bold* are the founding Histone Posse members).

When we (Beatrix “Trixi” Ueberheide and Sahana Mollah) joined the lab in the early 2000s, Don was already collaborating with the late C. David (Dave) Allis, a well-established chromatin scientist also at the University of Virginia at the time, leading to some initial publications (12, 13, 14, 15), and American Society for Mass Spectrometry conference presentations from former Hunt Lab members Jennifer Caldwell and Cynthia Brame as well (16, 17). These were exciting times and this new partnership resulted in the first instance of mass spectrometry helping identify novel histone modifications, such as arginine methylation (14), or several novel H2A C-terminal modifications (18). Dave was studying the role of histone PTMs in gene regulation, the emerging field of epigenetics, before it became the field as we know it now. In the early days, these studies were solely conducted with antibodies against specific histone modifications (19). Nevertheless, antibodies have limitations such as PTMs on nearby amino acids, which may hinder antibody binding, a situation called epitope occlusion, or cross-reactivity with another modification site (20). It was clear to Dave that an unbiased approach, namely mass spectrometry, would offer multiple advantages. The legend goes that Don and Dave had a conversation about histone PTMs 1 day in Don’s office, and the next day, Dave called Don because he was up all night and was excited because he was starting to figure out “the Code”! We all still remember the day when Dave Allis brought his entire lab to our group meeting and gave a passionate talk on how the modifications on histone proteins create a code that either turns the underlying genetic information on or off and how mass spectrometry was going to become a central tool for epigenetics research. At the end of his talk, Jeff Shabanowitz (Don’s right-hand scientist) stood up and proclaimed, “Anyone who is not motivated by this should not be in science and should leave the lab!”. We usually do not need our collaborators to try to convince us that their project is worth pursuing, but then our other collaborators did not bring us proteins that are extremely basic and a real challenge to analyze by standard mass spectrometry techniques (5, 6, 8). This is about the time when Ben Garcia decided to join the efforts on histone modifications. Ben remembers being worried to ask Don permission to essentially drop his current research projects to focus on histones, “What would Don say or how would he react?” However, Don was his usual calm self and told Ben, “I think that would be a wise choice.” Don was always supportive of his students (the Hunt-lings) and wanted them to get the most out of their lab experience. His number one priority was always his lab members, and he had no shortage of collaborator projects he thought were all great challenges ready for us to take on. Thus, the second generation of Hunt Lab members that would work on histone projects (the “Histone Posse”, Fig. 1) was formed with Sahana, Trixi, Ben, Celeste Ptak, and Scott Busby working round the clock to not only figure out how to analyze these very difficult proteins (develop new methodologies), but also to discovery new aspects of the Histone Code as well.

## The Grand Challenge

In theory, histone PTM analysis does not sound daunting at all. Histones are small proteins ranging in size from 11 to 16 kDa. Due to their highly basic nature, they can be easily isolated using a nuclear prep followed by TCA precipitation that yields very pure samples (>98% histones). Histones have so many positive charges that they are essentially the only nuclear proteins soluble in dilute sulfuric acid and, therefore, once precipitated, are isolated in high purity and yield. This fact resolves many typical issues related to isolating the specific group of proteins one is interested in studying from the cell prior to analysis on the mass spectrometer. Nevertheless, while easy to isolate, a histone sample can contain hundreds of modified forms (proteoforms) (21), each bearing varying combinations of modifications, resulting in some of the most highly modified and complex proteins in the human proteome. Currently, hundreds of modified sites on histones have been discovered by mass spectrometry and other approaches (22), validating histones as incredibly complex proteins to characterize and quantify by mass spectrometry approaches.

**Fig. 2:**
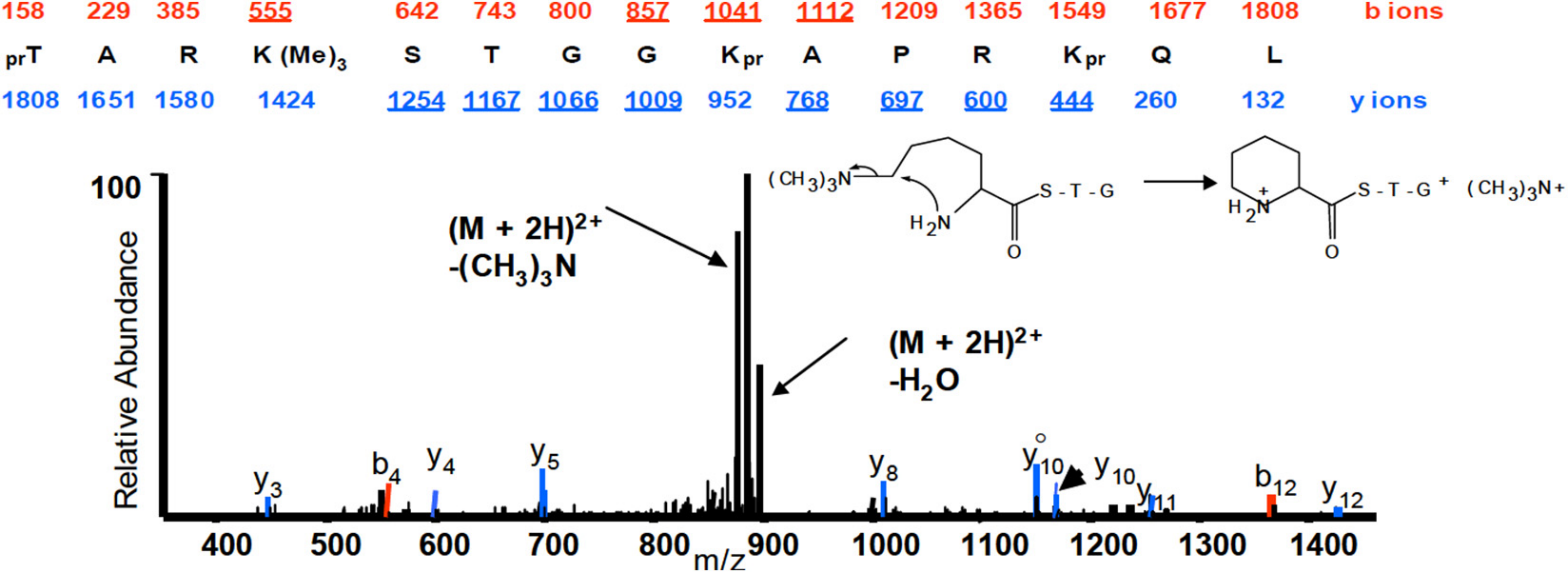
**MS/MS spectrum of the 6 to 20 residue fragment of histone H3.** Propionylation of this histone H3 peptide facilitates the analyses by converting the N-terminus and both K14 and K18 residues to propylamides (pr, +56 Da), while the K9 residue is not chemically modified as it is endogenously blocked by trimethylation. The peak at [M+2H]^2+^ −29.5 m/z corresponds to the loss of the trimethyl group (CH3)3N from the K9 residue (mechanism depicted). Adapted from Mollah *et al.* (28).

An example of this complexity can be found by considering histone H3 processed by chymotryptic digestion derived peptide spanning residues 6 to 20, TARKSTGGKAPRKQL (Fig. 2). This peptide has three lysine residues that may undergo acetylation, mono-, di-, or tri-methylation, two arginine residues that could be mono- or di-methylated, and two threonines and one serine residue that could be phosphorylated. Consequently, the possibility exists for eight concurrent or varying modifications on this small 15-mer peptide. Theoretically, there could be millions of combinatorially modified histone peptides. Besides the combinatorial explosion, histone proteins have the majority of PTMs at their N-termini. Unfortunately, for histone H3 and H4 specifically, every 3–4th amino acid on the N-terminus is either a lysine or an arginine. This, of course, makes the typical first step of proteomics, which is trypsin digestion, useless, as H3 and H4 proteins would be cleaved into peptides too small to be retained on C18 columns and analyzed by LC-MS/MS. Furthermore, modifications on lysines and arginines would interfere with trypsin and result in generating non-uniformity of digested peptides depending on their modification status, making quantitative work nearly impossible.

Not only were the points mentioned above making the analysis difficult, but the abundance of basic amino acids resulted in highly charged peptides (>+3). Back when we started this work in the early 2000s, the instrument of choice in the Hunt Lab was a Thermo LCQ ion trap mass spectrometer with collisional activated dissociation (CAD), electron transfer dissociation (ETD) (23), was not yet invented. This meant that we had neither high resolution nor the orthogonal ETD fragmentation at our disposal to a) identify the charge states easily and b) fragment highly charged peptides well. Moreover, proteomics data search engines back then could not easily handle the large number of concurrent modifications they barely can now. But the latter, being in the Hunt Lab, was a minor holdback as we were trained to manually confirm a spectrum, or else Don wouldn’t believe it. Therefore, we soldiered on and started to analyze this data manually with a deck of printed-out spectra, a ruler, a pencil, and a calculator, in true Don Hunt style. To this day we all still know where each major modified histone peptide elutes in relation to each other and what their major fragment ions are. Nevertheless, despite all these challenges, our first attempt at analyzing histone PTMs was quite successful (15, 24, 25). We first analyzed a histone sample using ArgC and Chymotrypsin digestion. ArgC was never quite efficient in our hands, and Chymotrypsin was not ideal either (it cut everywhere). Chymotrypsin was challenging to employ because it was difficult to limit non-specific site cleavages as trypsin, resulting in a complex soup of non-consistent peptides. We would be working on manually interpreting the MS/MS spectra, and it was common to spend all day sequencing a few peptides only to find out that they were the same modification site on a slightly different peptide. This was tedious but it would work for qualitative studies, which was the main focus in the early days of histone PTM analysis. "We remember Don coming in and asking if we had found any new PTM sites. Every once in a while, we did find some, such as histone H3T3phos (26), or H3K36ac (27). He would smile and give us the thumbs up after first inspecting the MS/MS himself! There was so much excitement when we all sat around Don’s office or in the lab and had phone calls with Dave Allis and his lab members to discuss the new data we generated from their samples (this was way before Skype or Zoom virtual meetings).

## Don to the Rescue

Nevertheless, a critical problem loomed and made histone PTM analysis difficult: the high basicity of these peptides. When using positive electrospray ionization (ESI) mode, the abundance of basic amino acids resulted in highly charged precursor ions (>+3), leading to the generation of multiply charged MS/MS fragment ions. Charge state deduction of the precursor ion and processing these MS/MS spectra was cumbersome and challenging given the limitations of the low-resolution LCQ and later available LTQ ion trap mass spectrometers. The hydrophilic peptides also did not retain well on C18 columns, another challenge as peptides were lost or all eluted out together. Adding to the complexity is the existence of isobaric species, where identical modifications can occur at distinct peptide sites, rendering them virtually indistinguishable through chromatographic means or accurate mass measurements alone. A simple example is a case where a histone peptide could be modified with a mixture of trimethylation and acetylation. While nowadays the differentiation is trivial, however, with low-resolution mass spectrometers, we had to rely on retention time shifts and diagnostic MS/MS ions. Trimethylated peptides, for example, eluted earlier than their acetylated counterpart due to being more hydrophilic and also lost part of the side chain upon CAD fragmentation (Fig. 2). To address the hydrophilicity issue, we conducted experiments involving an alternative ion pairing agent, heptafluorobutyric acid (HFBA), aimed at enhancing peptide retention on the column. While we did observe improved retention, better separation of peptides, and increased signal intensity for hydrophilic peptides, the hydrophobic peptides suffered from reduced intensity due to ionization suppression caused by HFBA. Thus, we abandoned this idea fairly quickly. Then Don came up with the million-dollar idea after a few attempts. Being an organic chemist by training, Don suggested we convert the lysine side chains into acetyl groups using acetic acid anhydride. Since the final result would exactly look like an endogenous acetylation, this had to be performed of course with “heavy” acetic anhydride. We decided to purchase deuterated (D6) acetic anhydride. This seemed to be the perfect solution, as the mass shift was 45 Da and thus, we should easily differentiate between endogenous (+42 Da) and our labeled *in vitro* acetylation, even with a low-resolution mass spectrometer. This derivatization had the extra benefit of reducing the hydrophilicity of these peptides and blocking a trypsin cleavage site. This effectively turned the tryptic digest into an efficient ArgC-type digestion, producing more uniform peptides while reducing enzyme costs. However, we discovered quickly that the deuterated reagent consistently contained a small percentage of contaminating D0 acetic anhydride, thus complicating our analysis by introducing false positive acetylated peptides. So, we all went back to the drawing board in Don’s office on a Friday to brainstorm on an alternative labeling reagent. This resulted in Don’s next and winning idea for our final protocol that has stood the test of time, and is still the standard in the histone proteomics field: the propionic anhydride derivatization, first revealed at ASMS 2002 (28), and later published as a protocol paper (29).

This propionylation derivatization of histones did it all. The reaction reduces peptide basicity and introduces a 56 Da hydrophobic moiety to every free N-terminus, unmodified, and monomethylated lysine residues. Consequently, this approach increases the chromatographic retention time on a reverse-phase column and simplifies the interpretation of MS/MS spectra by generating tryptic-like histone peptides. The histone peptide ions, now predominantly doubly and some triply charged, generate mostly singly charged fragment ions after MS/MS fragmentation using collisional methods. Moreover, since all lysine residues are either endogenously or exogenously modified, trypsin will no longer cleave at lysine residues, thereby preserving proteolytic cleavage consistency across various histone protein modification states. In simple terms, we were able to start quantifying histone modification sites more easily. With the 2002 ASMS Conference closing in on us and our abstract already submitted, of course, filled with overpromises and claiming we had fully developed a new method, the pressure was on and high. Would we turn out to be “Heroes or Zeroes?”, this was always the question of the week as we rode the histone highs and lows of scientific progress (or lack thereof). Everyone, including Don, was on edge, crossing our fingers that this method would completely work as we had hoped. To give an idea of how intense that time period was, we had a conversation with Don about trying out this new sample prep technique on a Friday, and less than 24 h later—Saturday afternoon—there we were, working away at our ion trap mass spectrometer. As we are collecting data, in walks Don, fresh from his tennis practice. He casually says, “Not that I expected you to be working over the weekend, but since you are, got any new data for me?”. To our relief, the method worked—and we had solid data for the ASMS conference! This became our go-to protocol, and not only did it work beautifully, but it’s also still being heavily used in labs worldwide today. That moment was a real turning point for us, and also demonstrated the keen thinking Don impacted on this problem that broke open the whole epigenetics-proteomics field in a way that no one expected.

## Impact of the Hunt Lab on the Histone Code

In the immediate years following the propionylation derivatization breakthrough, the Hunt Lab effectively employed this methodology in a number of histone studies conducted through nanoESI-LC–MS/MS using ion traps and then hybrid ion trap-Fourier transform instruments (26, 30, 31, 32, 33, 34, 35, 36, 37). These investigations have unveiled a range of novel modification sites within the N-terminal, globular core, and C-terminal domains of histones. Notable findings include differences in histone H3 and H1 variant modifications (26, 32, 34, 37), as well as quantifying histone marks under various biological conditions and human disease (30, 32, 33, 34, 35). A particularly exciting discovery from these initial Hunt and Allis Lab studies was the existence of the dual modification involving serine 10 phosphorylation and lysine nine methylation on histone H3 (36). Notably, this dual modification contradicted earlier *in vitro* HMTase assays, which had demonstrated the inability of synthetic peptides to be methylated at lysine nine when serine 10 was phosphorylated and vice versa (38). These new findings led to the refinement of the Histone Code and introduced the new “switch hypothesis” (36). According to the new framework, the position of the methyl mark influences whether phosphorylation serves as a regulatory 'on' or 'off' switch, thereby introducing an additional layer of control in gene repression and activation through histone PTMs. Interestingly, many common methylation marks on H3 (lysine 4, 9, and 27) are also direct neighbors of potential phospho marks (threonine 3, serine 10, and 28). Another study from the Hunt Lab subsequently revealed that these other methyl-phospho marks did indeed exist together, such as H3K27meS28phos and T3phosK4me as well (26).

The Hunt and Allis labs were an ideal productive partnership, and led to incredible new heights for the histone field over the years (12, 13, 14, 15, 24, 25, 26, 27, 31, 32, 33, 34, 36, 37, 39, 40, 41, 42, 43, 44, 45, 46, 47). The mapping and characterization of histone post-translational modifications (PTMs) have advanced rapidly since the early Hunt Lab breakthroughs. Mass spectrometry has been a total game-changer in the epigenetics field, leading to several new aspects of histone research. Thanks to newer mass spectrometry technologies—especially high-resolution platforms and the aforementioned ETD, we’ve moved well beyond simple identification and mapping. We can now perform both quantitative analysis, measuring the exact levels of specific modifications, and also middle and top-down analysis, which allows us to study entire intact histone proteins (22, 33, 41, 48, 49, 50). ETD in particular has revealed novel aspects of the Histone Code, especially the combinations of PTMs that occur simultaneously on large stretches of the histone protein. These approaches give us a clearer picture of how specific histone modification patterns and their quantities are linked to biological processes, helping us better understand their roles in gene regulation and chromatin structure. It is amazing to think how far we have come as a field in analyzing these proteins and that now they are no longer considered the mission impossible tasks they once were. Don, with his foresight and understanding of organic chemistry, as he frequently points out (which sadly none of us graduate students had), made one small but crucial adjustment that completely changed the Histone Code (51), and several scientific careers forever.

## Conflict of interest

The authors declare that they have no conflicts of interest with the contents of this article.
